# Sanitary Artificial Dentures

**Published:** 1905-03-15

**Authors:** N. S. Hoff


### SANITARY ARTIFICIAL DENTURES.

BY N. S. HOFF, D.D.S.

Read before the Southwestern Michigan Dental Society.

The occasion for this paper is not an overpowering conviction that it is a long overdue message that ought to be delivered to this particular audience in preference to some other, but is a natural sequence following an order from a superior officer to present a paper to this audience on some subject of value and interest at this time. The order came so peremptorily, and with no previous warning, that we felt exceedingly concerned to select a topic that would interest, to say nothing of edifying. Two or three aggravating cases of unsanitary mouths presenting themselves about the time our efforts to evolve by some mental exercise, of a vigorous character, a theme for this occasion, helped us to elect this one for your consideration. If it should seem inappropriate or its treatment prove inadequate, your forbearance is asked because your officers have given me but a few hours in which to formulate the matter for presentation.

The first thought to which I wish to call your attention, is the almost criminal neglect of oral sanitation by people generally. People who are scrupulously careful to have their faces and hands clean, their shoes blacked, their clothing well brushed, and other parts of the adornment in at least a fairly good state of cleanliness seem to forget their "false teeth" altogether, at least they neglect to give them a proportionate amount of attention.

We recently had a call from a very cultured gentleman on a professional matter, and were very much surprised to see the condition of a partial plate he was wearing. There was conclusive evidence that this man had no idea that it was incumbent upon him to take into account his plate when making his ordinary toilet. The condition was so disgusting as to render it practically impossible to render a proper service without first thoroughly disinfecting the whole mouth as well as the operative field.

One of the most common causes of unsanitary surroundings, resulting sooner or later in pathologic conditions, is a poorly-adapted or fitted plate. This is especially aggravating if it be a miserably-finished rubber plate for a partial case. You have all seen such plates which because they do not fit close to the teeth, or because of very thick borders, they provide spaces, or pockets into which all the refuse of the oral cavity gradually gravitates, and remaining undisturbed soon becomes a hotbed for growing myriads of disease-producing organisms. The blame in such cases does not rest wholly with the patient, as in many such cases it is practically impossible for the patient to keep his plate clean even if he were ever so well disposed. The man who made the plate was a *rascal*, and did only so much work on the plate as he was compelled to do in order that he might collect a fee for it, and of course he had no time or inclination, even if he possessed the ability, to instruct the patient how to care for it. He certainly did not measure up to the standard of the true professional man, who gives himself wholly to ministering in the best possible way to such as may place confidence in his skill and professional integrity. A clumsily-constructed artificial denture of any kind is an abomination, and the dentist who is not able to do this work skillfully had much better not try to do it at all, but confine himself to such work as he may do acceptably. Some one has said that three out of five dentists ought to take a post-graduate course in prosthetic technics. While this proportion is probably large, there undoubtedly are many practitioners who have no taste for this kind of work and never do a creditable piece of work. The queer thing about it all is that most of them think they can make plates as well as anybody. They may be able to make plates as fast as anybody, but they do not and many of them will never learn to adapt a useful and harmonious prosthetic appliance.

Next to the badly-constructed plates are the plates which do not fit the mouth as they should. Plates are often worn by patients that do not at all fit the mouth. They perhaps never did fit as they should, but more frequently they have been worn longer than they should have been. In some cases the mouth changes rapidly and a plate may become a misfit in a short time, such cases are usually reported and the matter adjusted with a new plate. But in many cases patients have worn a plate for ten or fifteen years and the gradual absorption which has been going on has given the patient time to become accustomed to the changes. The lack of adhesion which kept the plate in place, because of close adaptation, is not missed, because the patient learns to use the tongue and lip and buccal muscles to keep it in place while talking and eating. It is remarkable how expert some people become in this matter. It is, however, always at the expense of proper function, of distinct articulation and adequate mastication. In some cases it is a real trial to sit at table with a person who is so intent on controlling a pair of badly-fitting plates, that he either eats little or nothing, or disgusts everybody with his clatter and facial contortions. We know a very nice old gentleman who would be a very agreeable guest if he only had a set of teeth that did not need so much attention that the man can not carry on a conversation of any length without great embarrassment. I have often wanted to invite him to come to me for professional service, but have not had the courage to do so. Such a case—and there are many as bad, and possibly some worse—is not only a great misfortune from the social standpoint, but it is even more to be deplored from its sanitary aspect. The oral structures can not be healthy in such a mouth, because of the continual irritation, and because of the filth accumulations that must inevitably accompany it. No dentist is justified in compelling a patient to put up with such a condition of affairs. If it is the patient's fault, it seems to me that it would not be a breach of good manners or professional etiquette to remonstrate with such people and show them the bad effects that are sure to follow such a condition, and earnestly urge them to change the condition to one that will bring personal comfort, and lessen the menace to public health as well as good manners.

Then there are the old plates, which have been worn for thirty years—who would think of wearing a shirt or a pair of boots for thirty years—these old plates have often been repaired three or four times, and have so degenerated, structurally, that they are little better than so much punky wood. They are simply saturated with accretions of ages. Can they be healthy, even though the patient can masticate his food satisfactorily with them? How easy it is to refit such plates with new vulcanite or other material and without molesting the occlusion or form of plate. By this means a new and wholesome plate which can be made to fit the mouth perfectly can be substituted at small expense and great advantage to the patient. How many dentists feel called upon to look after their patients' interests in such a way? Most of us are so busy repairing damages that are imperative, that we overlook these cases, and patients do not realize that they should be given any attention. The patients are not so much to blame as the dentists for much of this condition. Patients think that as long as they can manage to masticate food, their plates are all they ought to be. This comes from the fact that dentists are accustomed to tell patients that the work they do is permanent; meaning that the life of the filling, crown or plate is indefinitely a long one. Patients frequently inquire when arranging for work if the dentist warrants his work—indicating that they have an idea that it should last a long time. Perhaps the most inexcusable and prolific cause of pathologic, or at least unhealthy, conditions of the mouth is to be found as a result of patients wearing even new and well-adapted plates the palatal surfaces of which have not been properly finished. Many dentists seem to have an idea that no attempt should be made to finish the palatal surface of rubber plates for fear that some of the detail will be removed and the adhesion of the plate be lost. It is not uncommon to find not only the plate left just as it came from the vulcanizer, but exceedingly rough, caused by the fact that the model was poorly made and no effort at all has been made to provide a hard and smooth surface upon which to vulcanize. This surface may also be further mutilated with deep suction chambers and other mutilations to increase its irritating capacities.

We appreciate the fact that it would be a difficult and laborious task to undertake to polish the entire palatal surface of an eight-dollar rubber plate, so that it would be as smooth and comfortable as a gold plate. This may suggest the idea that a dentist has no right to inflict eight-dollar rubber plates on his patients. It may be that he would feel justified in receiving a sufficient fee from a grateful patient should he take the trouble to explain to his patient the value of such an additional expense. There is really no excuse on such grounds for much of the discreditable work one frequently sees. If reasonable care be taken to secure good models, and before packing the vulcanite, proper preparation of the surface of the model with tin foil or liquid silex be made, the surface of the plate will come from the vulcanizer comparatively free from irritating projections, and it can be polished without much work so that an agreeable, smooth, hard surface will be presented to the mucous membrane. And what is of prime importance, a surface will be provided which will not invite accumulations of the food-stuffs or vitiating mouth secretions.

Much had been said and written about the deleterious action of the mercuric coloring matter of red rubber in producing stomatitis and other affections of the oral mucous membranes. The probabilities are that little or none of the unwholesome oral conditions are due to this cause.

The mechanical and structural defects are amply sufficient to cause great disturbances, and in some cases it does seem as though the oral tissues are wonderfully immune and resistant, or they would certainly succumb to the continued mechanical and unsanitary irritations to which they are subjected.

Rubber plates are too often inadequately vulcanized. They are made thick and clumsy with little or no regard to the comfort of the patient and not much conception of the stress to which they will be subjected in use. Then they are vulcanized in the shortest possible time and insufficiently to produce a hard and dense plate which will not absorb the secretions in which they are continually bathed. Many times the plates have degenerated under the rapid and excessive application of the heat, producing apparently perfect plates on the surface, but within a sponge, and in case no proper care has been taken to provide constant spring pressure, while vulcanizing the rubber, draws away from the teeth while hardening, leaving spaces which soon fill up with secretions to ferment and irritate the mouth, and it may be to cause a nauseating odor which no ordinary disinfection or cleansing will eradicate.

We have not enumerated all the causes, or the details of any, which go to make up the defects in artificial dentures as most generally made at the present day; and it may be that as long as this subject remains on the present commercial basis, and the better men in the profession do only so much of this work as they are compelled to do, and because of lack of practice or knowledge, they do it in so unskillful a manner, there may be no remedy. Intelligent people if made acquainted with the consequences of inferior work along this line, would certainly acquiese in good work and pay reasonable fees. It seems as though it were high time this branch of our profession was receiving some attention from those who are capable of doing a skillful piece of work and have some conception of how such work ought to be done to secure the best results. Every other department of practice seems to have made progress except this, and it has appreciably degenerated in the last twenty-five years. It may be that sufficient interest can be worked up from the standpoint we here present. It is very apparent that little or no improvement can be looked for from the mere technical aspect. This department seems to be drifting through the commercial laboratory and hired "*technicker*" entirely out of the hands of the educated dentist. That such a result should come to pass would be deplorable enough, and the only way to stop this tendency is for the profession to realize the importance of the application of all accumulated sanitary knowledge as well as technical skill to this important department of dental practice. There seems to be a decided reaction from the conspicuous and inartistic gold crown and bridge-work, to more esthetic and sanitary practice; may we not hope and work for a reform of the present ideas and practices in regard to plate-work. "It is a consummation devoutly to be wished."

